# Age-dependent differential regulation of anxiety- and depression-related behaviors by neurabin and spinophilin

**DOI:** 10.1371/journal.pone.0180638

**Published:** 2017-07-10

**Authors:** Huiying Wu, Christopher Cottingham, Liping Chen, Hongxia Wang, Pulin Che, Kexiang Liu, Qin Wang

**Affiliations:** 1 Ultrasonic Diagnosis Department, The Second Hospital of Jilin University, Changchun, Jilin, China; 2 Departments of Cell, Molecular and Developmental Biology, University of Alabama at Birmingham, Birmingham, AL, United States of America; 3 Department of Biology and Chemistry, Morehead State University, Morehead, KY, United States of America; 4 Department of Cardiovascular Surgery, The Second Hospital of Jilin University, Changchun, Jilin, China; Augusta University Medical College of Georgia, UNITED STATES

## Abstract

Affective disorders impact nearly 10% of the adult population in the United States in a given year. Synaptic dysfunction has recently emerged as a key neurobiological mechanism underlying affective disorders such as anxiety and depression. In this study, we investigate the potential role of two synaptic scaffolding proteins, neurabin and spinophilin, in regulating anxiety- and depression-related behaviors at different ages using genetically deficient mice. Loss of the neurabin gene reduces anxiety-like behavior in the elevated zero maze in young adult mice (3–5 months old), but not in middle aged mice (11–13 months old), whereas loss of spinophilin decreases anxiety in middle-aged mice, but not in young adult mice. Neurabin knockout (KO) mice also show reduced immobility in the repeated force swim test (FST) at 3–5 months, but not 11–3 months, of age, compared to age- and strain-matched wild type (WT) controls. Conversely, spinophilin KO mice display a lower level of this behavioral despair than matched WT controls after repeated FST trials at the middle age (11–13 months) but not the young age (3–5 months). Together, these data indicate that, despite their structural similarities and overlapping function in regulating synaptic cytoskeleton, the two homologs neurabin and spinophilin play important yet distinct roles in the regulation of anxiety- and depression-like behaviors in an age-dependent manner. Our studies provide new insights into the complex neurobiology of affective disorders.

## Introduction

Affective disorders, such as anxiety and depression, are a major public health challenge carrying substantial social and economic burdens, impacting nearly 10% of the adult population in the United States in a given year [1]. Despite extensive efforts in recent decades, we continue to lack a coherent view of the neurobiology of affective disorders, which limits the development of efficacious therapeutics. However, disruption of synaptic function, including both connectivity and plasticity, has recently emerged as a key neurobiological mechanism underlying affective disorders [2–4]. Dysregulation of synaptic proteins often occurs in these conditions, and may contribute to affective disorder etiology.

Neurabin and spinophilin, which are also referred as neurabin I and II, are homologous scaffold proteins enriched at the synapse. The two proteins share a similar multi-domain structure which includes actin-binding, G protein-coupled receptor (GPCR)-interacting, protein phosphatase 1 (PP1)-binding, PDZ, and coiled-coil domains [5, 6]. Neurabin also has a unique SAM domain at the C-terminus [5]. Neurabin is localized to both pre- and postsynaptic compartments [7] and is required for presynaptic assembly during development [8]. Spinophilin, by contrast, primarily localizes to the postsynaptic compartment, specifically dendritic spines [6, 7, 9]. Both neurabin and spinophilin have been shown to modulate spine morphogenesis and maturation through regulation of the actin cytoskeleton [10–15]. In addition, these two scaffolding proteins can target PP1 to AMPA receptors in postsynaptic densities [16, 17], regulating their trafficking [10, 17] and affecting AMPA receptor-mediated fast excitatory synaptic transmission. Neurabin and spinophilin also directly interact with multiple GPCRs, and regulate receptor trafficking and signaling [18–24]. Such regulation can, in turn, lead to changes in synaptic plasticity [24, 25].

Given their functions in regulating synaptic morphology and plasticity, we elected to address the potential roles of neurabin and spinophilin in regulating anxiety and depression-related behaviors using neurabin-deficient and spinophilin-deficient mice. Since these behaviors are often affected by age, we tested two age groups, young adult (3–5 months old) and middle aged (11–13 months old), in the open field (OF), elevated zero maze (EZM), and forced swimming test (FST) assays. Our data reveal that, despite the structural similarities between neurabin and spinophilin, and their overlapping function in regulating the synaptic cytoskeleton, inactivation of each gene caused distinct behavioral phenotypes. Furthermore, the phenotypes are age-dependent for both neurabin and spinophilin, although the overall expression levels of these two proteins did not change with age. Our study suggests that neurabin and spinophilin play important, yet distinct, roles in regulating anxiety- and depression-like behaviors in an age-dependent manner.

## Methods and materials

### Animals

Mice were housed in the Association for Assessment and Accreditation of Laboratory Animal Care-accredited Animal Resources Program at the University of Alabama at Birmingham. Experimental procedures are in accordance with Animal Welfare Act and the 1989 amendments to this Act. All protocols were approved by University of Alabama Institutional Animal Care and Use Committee. Mice were euthanized by CO_2_ inhalation followed by cervical dislocation to ensure the death of the animals. CO_2_ flow was controlled by a regulator. The procedure was consistent with the recommendations of the AVMA Guidelines for the Euthanasia of Animals.

Neurabin-null [25] and spinophilin-null [26] mouse lines were generated previously. These lines were backcrossed to the C57BL/6 genetic background for more than 12 generations and maintained on this background. Wild type (WT) C57BL/6, neurabin knockout (KO) and spinophilin KO mice were obtained from homozygous breeding pairs of each corresponding genotype. Male mice were tested at two different ages, 3–5 months old (young adult) and 11–13 months old (middle-aged).

### Open-field (OF) and elevated zero maze (EZM) tests

OF and EZM tests were performed as we described previously [27]. OF trials were conducted in an open field arena of area 42 cm^2^. Total distance traveled and relative time spent in the center of the open field were recorded. EZM was an elevated cycling corridor with two open areas and two closed areas. Time spent in open versus closed areas of the EZM and total distance traveled were documented as endpoints. All data were acquired using the automated EthoVision camera-driven tracker system (Noldus, The Netherlands).

### Forced swim test (FST)

FST were performed as previously described [27] with modifications. Mice were individually placed in a glass cylinder (25 cm x15 cm diameter) filled with room temperature water and videotaped from the side for 6 min on day 1. The duration of immobility in the last 4 min was documented. On the second day, mice were subjected to three consecutive trials of FST at 1-hr intervals. Each trial was for 4 min and the duration of immobility over the entire 4 min was documented.

### Immunoblotting

Mouse brains without the cerebellum were homogenized with lysis buffer containing 75mM Tris, pH6.8, 15% glycerol and 3% SDS. 20μg of each sample was loaded on a 10% polyacrylamide gel and separated by SDS-PAGE. Neurabin, spinophilin and PP1γ were detected by antibodies against neurabin (Santa Cruz, H-300), spinophilin (Upstate) and PP1γ (Santa Cruz, C-19), respectively. Quantitation of Western blots was performed using the LI-COR Odyssey Imaging System.

### Statistics

Data were analyzed using GraphPad Prism software. Unpaired Student’s *t*-tests were used to compare two groups. Two-way ANOVA was used to analyze multiple FST trials with two genotypes and test for trial x genotype interactions in FST. Post hoc Sidak's multiple comparisons were performed following two-way ANOVA to determine the difference between two genotypes in each FST trial.

## Results and discussion

### Loss of neurabin, but not spinophilin, in young adult mice has an anxiolytic effect on behavior

**Fig 1:**
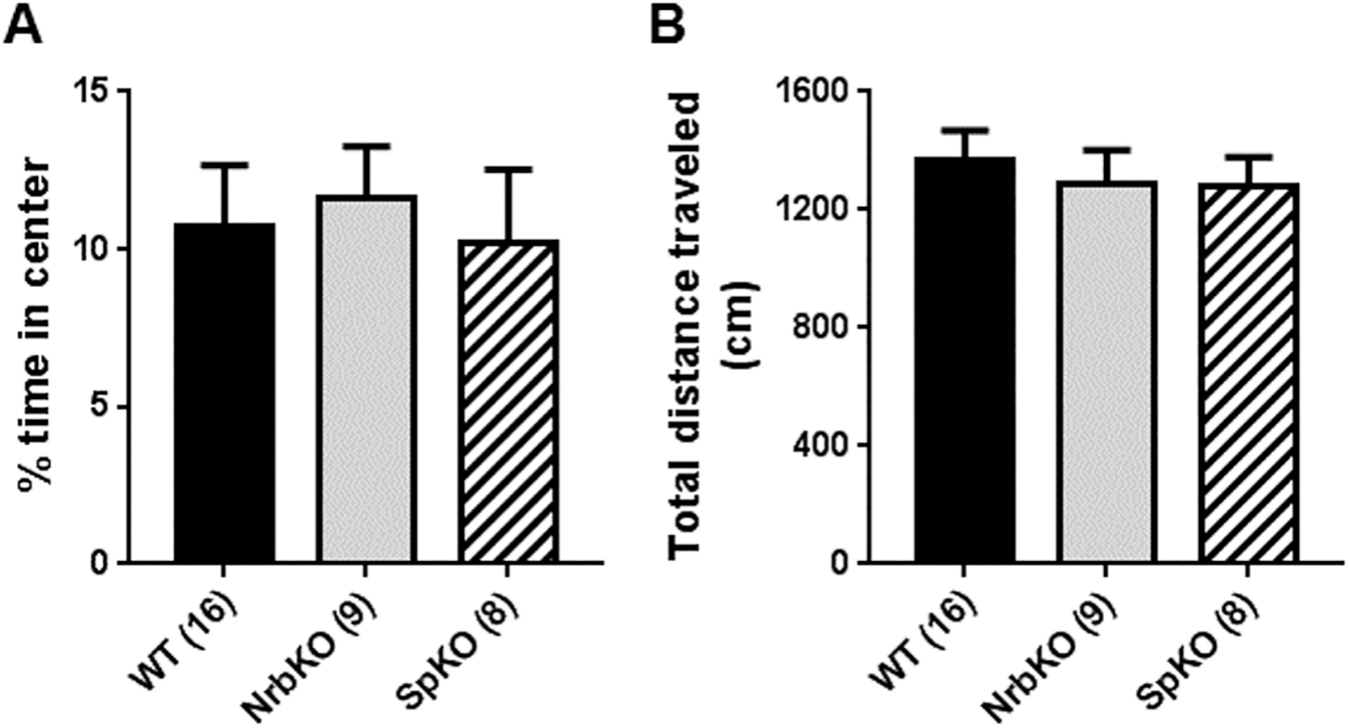
Open-field (OF) analysis of mice at 3–5 months of age (young adult). Age- and strain-matched wild-type (WT, n = 16) mice were evaluated in parallel with neurabin KO (NrbKO, n = 9) and spinophilin KO (SpKO, n = 8) mice. (A) The percent of time spent in center during the entire OF trial time (i.e., center exploration time). (B) Total distance traveled during the entire OF trial time. Data are mean ± SEM.

We began by assessing the baseline activity of young adult (3–5 months old) neurabin KO and spinophilin KO mice in the OF assay, comparing with age- and strain-matched WT mice. No difference was observed in either the percentage of time spent in the center of the arena (i.e., center exploration) over the total trial time (Fig 1A) or the total distance traveled in the arena (Fig 1B) for either KO group as compared to WT mice.

**Fig 2:**
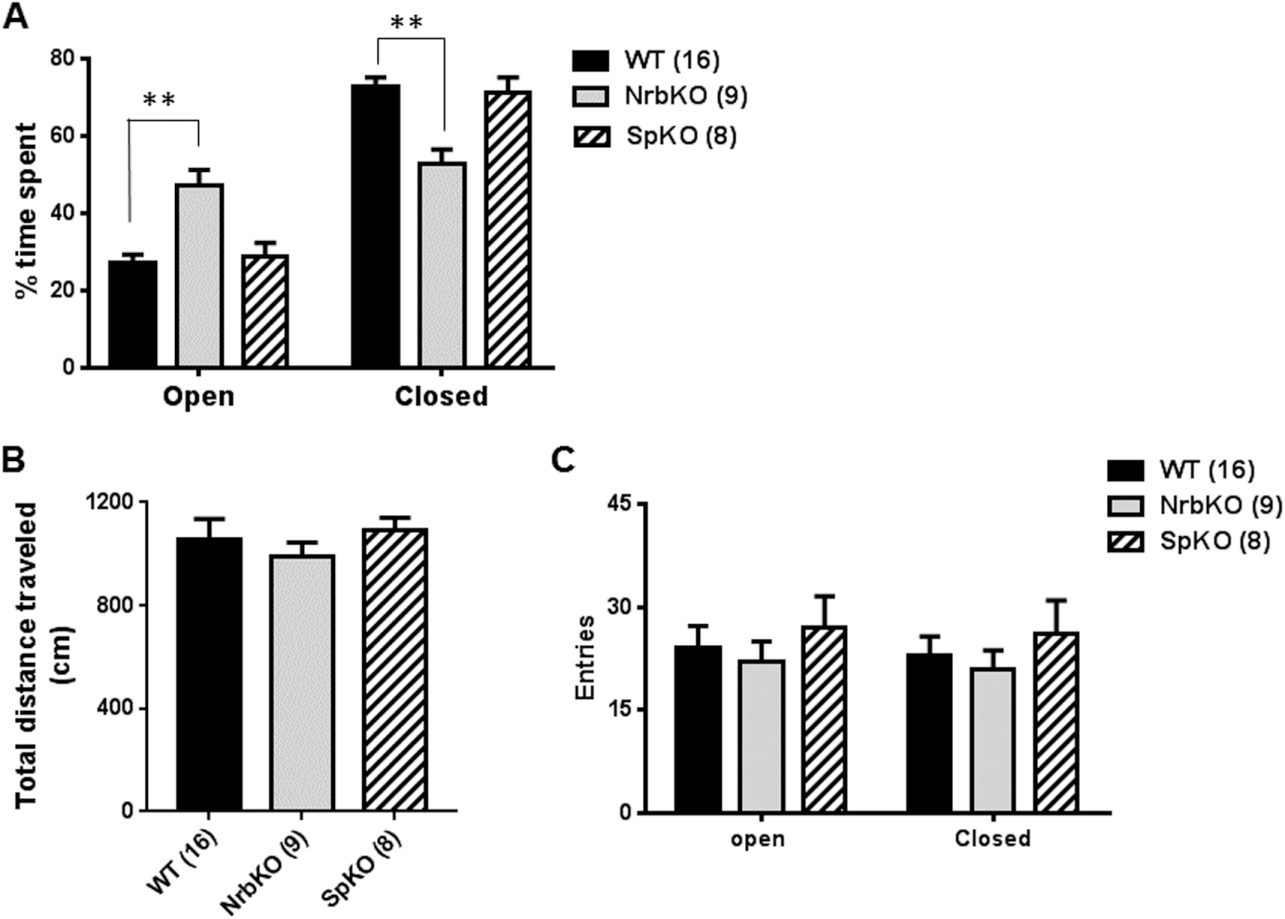
Elevated zero maze (EZM) analysis of mice at 3–5 months of age (young adult). Age- and strain-matched WT (n = 16) mice were evaluated in parallel with NrbKO (n = 9) and SpKO (n = 8) mice. (A) The percentage of time spent in the open versus closed regions of the EZM during the entire trial time. (B) Total distance traveled during the entire EZM trial time. (C) Entries into the open and closed area of EZM during the trial. Data are mean ± SEM. **, *p*<0.01, NrbKO *vs*. WT by Student’s *t* test.

We then examined anxiety-like behavior by EZM tests. Because of the elevated nature of the platform, rodents tend to spend more time in the closed regions than in the open ones. When comparing the effects of genotype, drug administration, etc., any increase in time spent in the open regions indicates decreased anxiety-like behavior, while any increase in time spent in the closed regions indicates increased anxiety-like behavior [28]. As shown in Fig 2A, both spinophilin KO and age- and strain-matched WT mice spent more than 70% of the trial time in the closed EZM regions, while neurabin KO mice spent an almost equal amount of time in the open and closed regions. The resulting statistically significant increase in open region time and decrease in closed region time suggests reduced anxiety-like behavior in neurabin KO mice, compared with the matched WT mice. Similar total distances traveled (Fig 2B) and numbers of entries into the open or closed regions (Fig 2C) were observed across all genotypes. Together, our data suggest that neurabin, but not spinophilin, is involved in the regulation of anxiety-like behavior in young adult mice, with the loss of neurabin having an anxiolytic effect.

Center exploration time in the OF is an additional parameter that can reflect anxiety-related behavior, with increased center time being indicative of an anxiolytic effect [29]. Although young adult neurabin KO (Fig 2A) mice exhibited reduced levels of anxiety-related behaviors compared to matched WT controls in the EZM assay, no alteration in OF center exploration time was observed for these animals (Fig 1A). Such a discrepancy has been reported previously with young adult neurabin KO mice [30]. This previous work and our current data collectively suggest that neurabin KO mice are selectively sensitive to the anxiogenic conditions in the EZM but not in the OF, with the anxiolytic effect of neurabin loss manifesting only in the former.

### Differential regulation of depression-like behavior in the FST by neurabin and spinophilin in young adult mice

**Fig 3:**
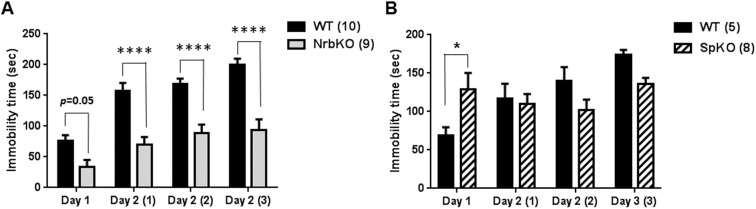
Immobility time of NrbKO and SpKO mice in the forced swim test (FST) at 3–5 months of age (young adult). A two-day repeated FST assay was performed as described in Materials and Methods. (A) Immobility time (in sec) in each trial for age- and strain-matched WT (n = 10) and NrbKO (n = 9) mice tested in parallel. (B) Immobility time in each trial for age- and strain-matched WT (n = 5) and SpKO (n = 8) mice tested in parallel. Data are mean ± SEM. *, *p*<0.05; ****, *p*<0.0001, compared to WT by post hoc Sidak's multiple comparison test following two-way ANOVA.

The FST has been widely used to evaluate behavioral despair [31, 32], typically considered a depression-like behavior in rodent models. To determine the roles of neurabin and spinophilin in regulating depression-like behavior, we performed a two-day repeated FST assay. On day 1, mice were tested in one 6-min trial with the immobility time in the last 4 min recorded. In this initial trial, young adult neurabin KO mice showed a trend toward reduced immobility compared with matched WT mice (*p* = 0.05, Fig 3A). While this appears to somewhat conflict with a previous report that neurabin KO mice show increased immobility time in the FST [30], the discrepancy may be attributable to a difference in genetic background. The neurabin KO mice in our study were on the C57BL/6 genetic background, and differences between mice strains are well known to exist for behavioral phenotypes such as those observed in the FST [33]. Different from neurabin KO mice, spinophilin KO mice displayed a significantly increased immobility time (Fig 3B) compared with matched WT mice. These data suggest that these two homologs play opposing roles in regulating behavioral despair at this age.

On day 2, mice were tested in 3 consecutive 4-min trials at1 hr intervals. Repeated forced swim stress can induce learned helplessness in rodents, thus serving as a further model for depression-like behavior [34, 35]. The immobility time of WT mice in the day 2 trials was dramatically increased compared to the initial day 1 trial (Fig 3A and 3B), reflecting an acquisition of learned helplessness by the mice. Although slightly increased compared to the initial day 1 trial, the immobility time of neurabin KO mice in all day 2 trials were markedly reduced compared to the matched WT mice (Fig 3A), indicating reduced acquisition of learned helplessness in these animals. This, in turn, suggests a phenotype of reduced depression-like behavior with loss of neurabin. Two-way ANOVA on the raw values revealed a significant trial x genotype interaction (*p* < 0.01) for immobility time (Table 1). Conversely, spinophilin KO mice failed to show a repeated trial-dependent increase in immobility time (Fig 3B), suggesting that these mice are insensitive to forced swimming stress. Two-way ANOVA on the raw values revealed a significant trial x genotype interaction (*p* < 0.01) for immobility (Table 2). Together, these data suggest that loss of neurabin leads to reduced depression-like behavior in young adult mice, and that neurabin and spinophilin play different roles in regulating depression-like behavior.

**Table 1:** Two-way ANOVA table for Fig 3A.

ANOVA table	SS	DF	MS	F (DFn, DFd)	P value
Interaction	19751	3	6584	F (3, 33) = 6.259	P = 0.0018
Row Factor (Trials)	20653	3	6884	F (3, 33) = 6.545	P = 0.0014
Column Factor (Genotype)	438.5	1	438.5	F (1, 11) = 0.1498	P = 0.7061
Subjects (matching)	32199	11	2927	F (11, 33) = 2.783	P = 0.0113
Residual	34711	33	1052		

**Table 2:** Two-way ANOVA table for Fig 3B.

ANOVA table	SS	DF	MS	F (DFn, DFd)	P value
Interaction	9893	3	3298	F (3, 51) = 5.918	P = 0.0015
Row Factor (Trials)	90098	3	30033	F (3, 51) = 53.9	P<0.0001
Column Factor (Genotype)	119100	1	119100	F (1, 17) = 31.45	P<0.0001
Subjects (matching)	64377	17	3787	F (17, 51) = 6.796	P<0.0001
Residual	28418	51	557.2		

### Loss of spinophilin increases baseline activity and has an anxiolytic effect in middle-aged mice

**Fig 4:**
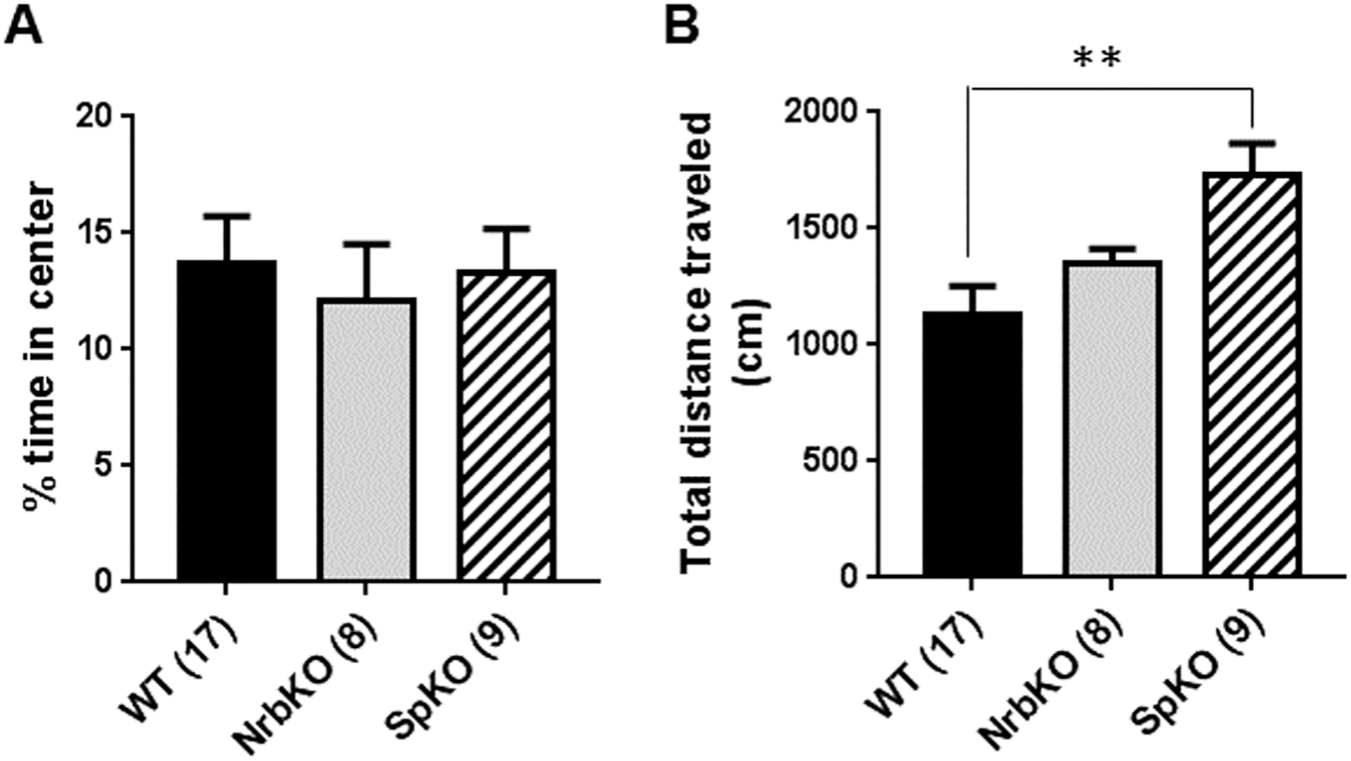
OF analysis of at 11–13 months of age (middle-aged). Age- and strain-matched WT (n = 17) mice were evaluated in parallel with NrbKO (n = 8) and SpKO (n = 9) mice. (A) The percent of time spent in center over the total trial time. (B) Total distance traveled during the OF trial. Data are mean ± SEM. **, *p*<0.01, SpKO *vs*. WT by Student’s *t* test.

It has long been observed that the occurrence of affective disorders tends to vary with age [36, 37]. In animal models, sensitivity to stress and antidepressant treatment are both often affected by age as well [38, 39]. Therefore, we sought to determine whether the behavioral alterations observed in neurabin KO and spinophilin KO mice change with age by evaluating mice of these genotypes in the above assays at 11–13 months old (middle-aged). We first examined their baseline activity in the OF assay. No difference was observed in center exploration time for either neurabin KO or spinophilin KO mice as compared to matched WT mice (Fig 4A). However, spinophilin KO mice appear to be more active than WT, as reflected by an increase in total distance traveled in the arena for these animals (Fig 4B).

**Fig 5:**
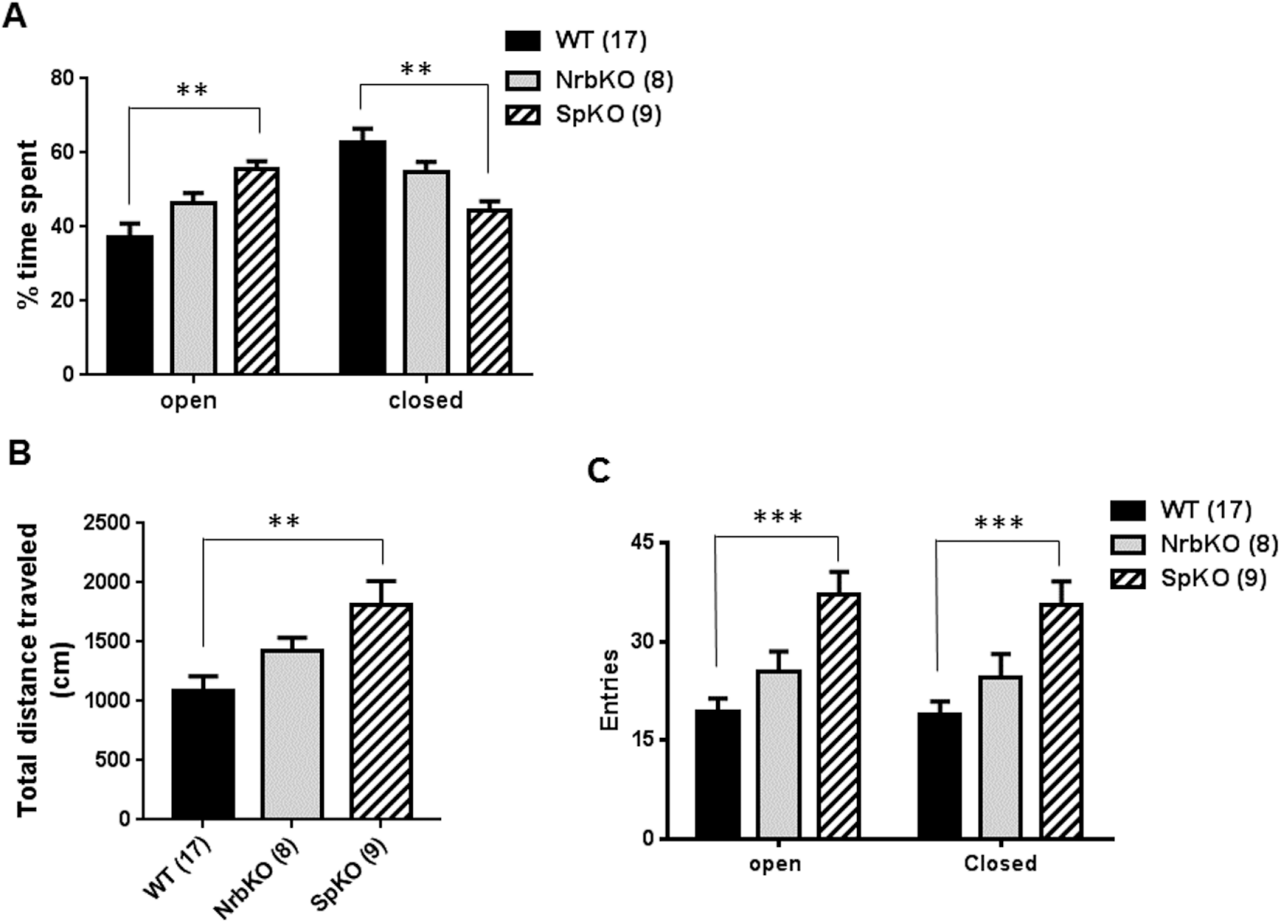
EZM analysis of at 11–13 months of age (middle aged). Age- and strain-matched WT (n = 17) mice were evaluated in parallel with NrbKO (n = 8) and SpKO (n = 9) mice. (A) The percentage of time spent in the open and closed area of EZM over the total trial time. (B) Total distance traveled during the EZM trial. (C) Entries into the open and closed area of EZM during the trial. Data are mean ± SEM. **, *p*<0.01; ***, *p*<0.001, SpKO *vs*. WT by Student’s *t* test.

We then analyzed the anxiety-like behavior for the middle-aged mice in the EZM. In this assay, spinophilin KO mice spent significantly more time in the open regions of the EZM, and less in the closed regions, compared with matched WT mice, while neurabin KO mice displayed no difference with WT (Fig 5A). These data indicate reduced anxiety-like behavior in the middle-aged spinophilin KO mice, suggesting an anxiolytic effect of spinophilin loss at this age. In addition, the anxiolytic effect of neurabin loss observed in the younger mice (see Fig 2) is absent in the older individuals. Furthermore, the increased activity level of the spinophilin KO mice was again observed in the EZM, as these individuals traveled a longer distance (Fig 5B) and had an increased number of entries into both the open and closed regions (Fig 5C) compared with matched WT mice. Taken together, these data suggest that, spinophilin, but not neurabin, is involved in the regulation of baseline activity and anxiety-like behavior in middle-aged mice.

### Neurabin and spinophilin regulate depression-like behavior during different phases of the FST in middle-aged mice

**Fig 6:**
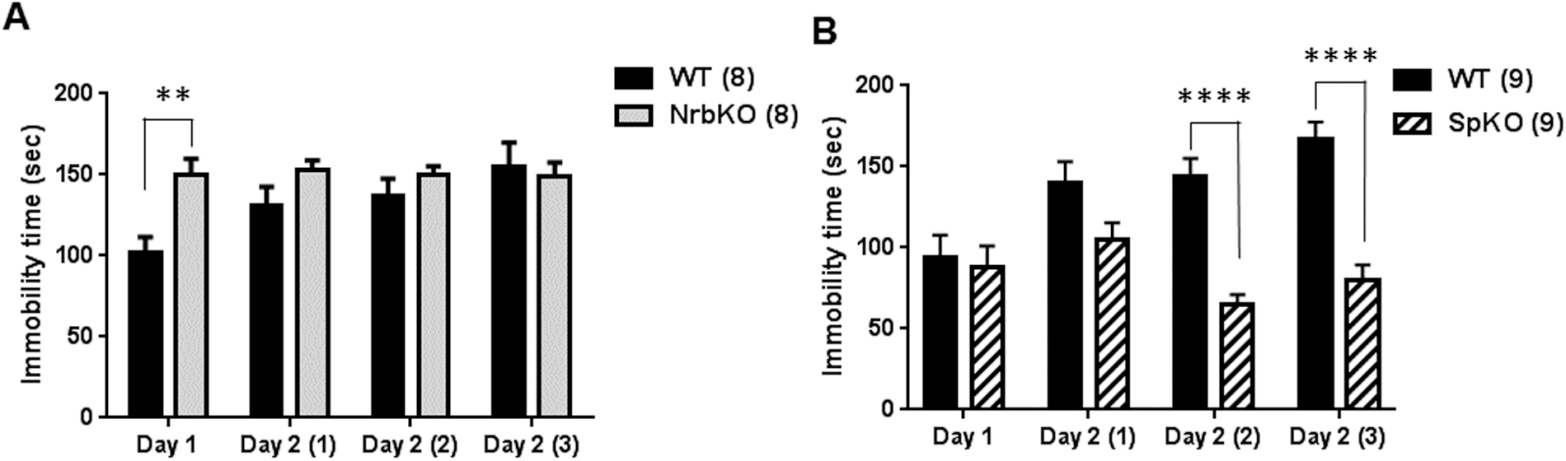
Immobility time of NrbKO and SpKO mice in FST at 11–13 months of age (middle-aged). (A) Immobility time (in sec) in each trial for age- and strain-matched WT (n = 8) and neurabin KO (n = 8) mice tested in parallel. (B) Immobility time in each trial for age- and strain-matched WT (n = 9) and spinophilin KO (n = 9) mice tested in parallel. Data are mean ± SEM. **, *p*<0.01, NrbKO *vs*. WT; ****, *p*<0.0001, SpKO *vs*. WT by post hoc Sidak's multiple comparison test following two-way ANOVA.

We further performed the two-day repeated FST assay on the middle-aged mice of different genotypes. Intriguingly, the reduced depression-like behavior in neurabin KO mice observed with young adults was lost in the middle-aged individuals (Fig 6A). In fact, neurabin KO mice showed significantly increased immobility time in the initial day 1 trial compared to matched WT mice (Fig 6A). In addition, these KO mice failed to show a repeated trial-dependent increase in immobility time (Fig 6A), suggesting insensitivity to forced swimming stress. Two-way ANOVA on the raw values revealed a significant trial x genotype interaction (*p* < 0.05) for immobility time between WT and neurabin KO mice (Table 3). These data suggest that, at the point of middle age, neurabin loss leads to an overall elevation in FST immobility time, such that no increases can be observed across the repeated trials.

**Table 3:** Two-way ANOVA table for Fig 6A.

ANOVA table	SS	DF	MS	F (DFn, DFd)	P value
Interaction	6168	3	2056	F (3, 42) = 3.557	P = 0.0221
Row Factor (Trials)	5669	3	1890	F (3, 42) = 3.27	P = 0.0304
Column Factor (Genotype)	6006	1	6006	F (1, 14) = 4.486	P = 0.0525
Subjects (matching)	18744	14	1339	F (14, 42) = 2.317	P = 0.0181
Residual	24273	42	577.9		

On the other hand, the immobility time in the initial day 1 trial for spinophilin KO mice was comparable to that of WT mice. As was observed for the young adult mice, no acquisition of learned helplessness behavior was found with repeated forced swim stress in the middle-aged spinophilin KO mice (Fig 6B). Furthermore, the immobility time in the last two trials on day 2 for spinophilin KO mice was significantly less than those for WT mice (Fig 6B). Two-way ANOVA on the raw values revealed a significant trial x genotype interaction (*p* < 0.0001) for immobility time between WT and spinophilin KO mice (Table 4). These data suggest that, different from the effect of neurabin deficiency, loss of spinophilin imparts an apparent protective effect in terms of the depression-like behavior of swimming stress-induced learned helplessness at this age.

**Table 4:** Two-way ANOVA table for Fig 6B.

ANOVA table	SS	DF	MS	F (DFn, DFd)	P value
Interaction	19695	3	6565	F (3, 48) = 15.88	P<0.0001
Row Factor (Trials)	12942	3	4314	F (3, 48) = 10.44	P<0.0001
Column Factor (Genotype)	48412	1	48412	F (1, 16) = 15.31	P = 0.0012
Subjects (matching)	50604	16	3163	F (16, 48) = 7.652	P<0.0001
Residual	19840	48	413.3		

**Fig 7:**
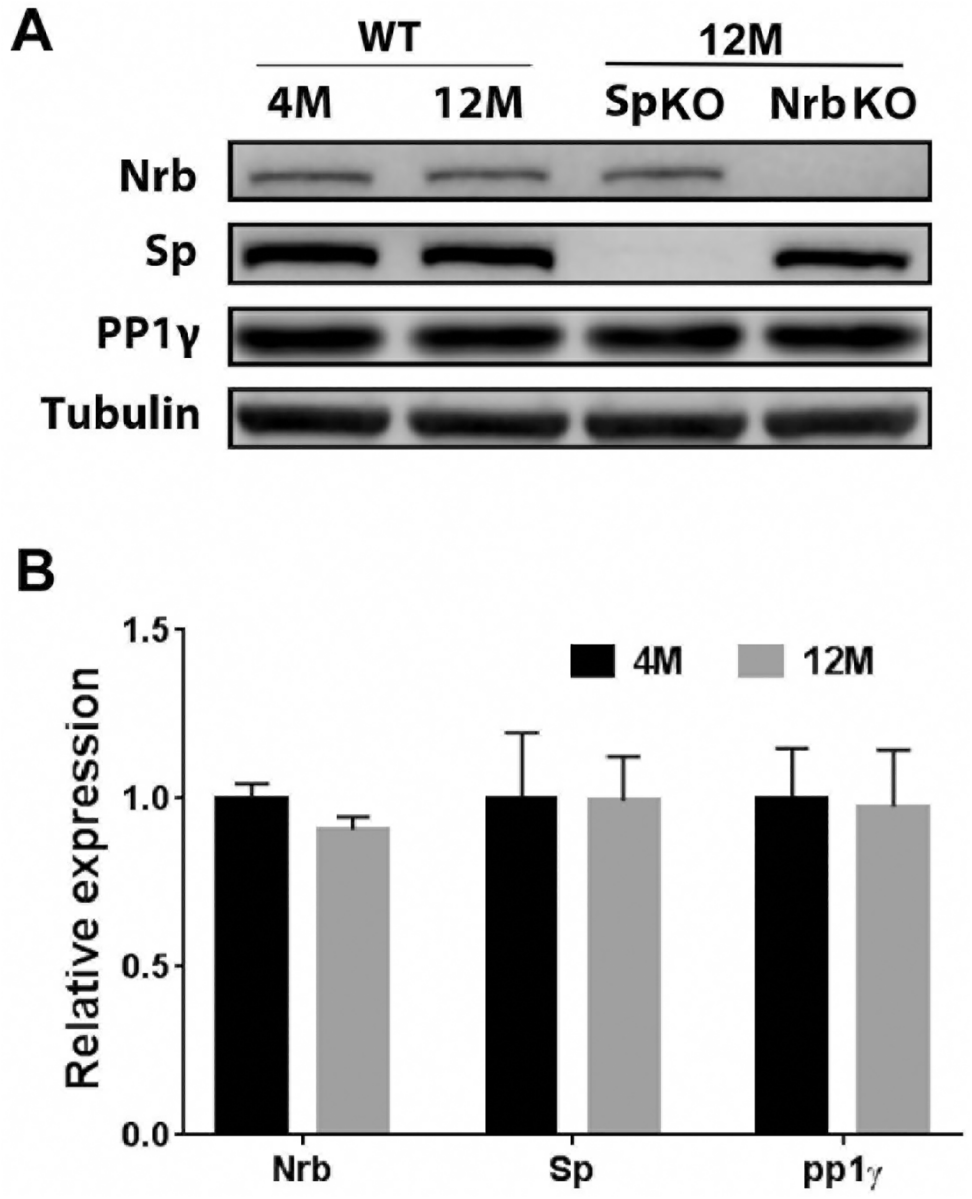
Expression of neurabin and spinophilin in the mouse brain at 4 and 12 months of age. Total brain lysates were subjected to western blot. Neurabin, spinophilin, PP1γ and tubulin (loading control) were detected by respective antibodies. (A) representative westerns. (B) Quantification of expression levels of neurabin, spinophilin and PP1γ in the WT mouse brain at different ages. Data are presented as mean ± SEM. n = 3 for each group.

Since we observed distinct phenotypes at two different ages for both neurabin KO and spinophilin KO mice, we further tested whether the expression level of these proteins changes at different ages. As shown in Fig 7, the overall expression levels of neurabin and spinophilin in the brain at 4 months of age were similar to those at 12 months of age. However, given that spinophilin and neurabin are differentially expressed in various brain regions ([6] and data shown in Allen Brain Atlas), brain region-specific changes in expression of these two proteins may occur at different ages. In addition, it has been reported that association of spinophilin with synaptic partners changes at different ages [40]. Such kind of age-dependent dynamic regulation may affect the abilities of spinophilin and neurabin to localize in postsynaptic density and regulate neural plasticity. Despite of their structural similarities and shared abilities to interact with both actin filaments and PP1γ, neurabin and spinophilin have been shown to play distinct roles in regulating long term plasticity at corticostriatal synapses [25]. While neurabin KO mice display deficient long-term potentiation but normal long-term depression, spinophilin KO mice show impaired long-term depression but normal long-term potentiation [25]. Differences in neural plasticity could underlie the expression of different behavioral phenotypes in these two KO lines.

In summary, our present study addressed the roles of two synaptic scaffolding proteins, neurabin and spinophilin, in regulating anxiety- and depression-related behaviors, using mice with each individual gene inactivated. In the EZM assay, loss of neurabin leads to a reduction in anxiety-like behavior in young adult mice (3–5 months old), indicative of an anxiolytic effect (Fig 2A), but not in middle aged mice (11–13 months old, Fig 5A). Meanwhile, loss of spinophilin reduces anxiety-like behavior in middle-aged mice (Fig 5A), again indicative of an anxiolytic effect, but not in young adult mice (Fig 2A). The two mouse lines also displayed distinct phenotypes in the two-day repeated FST assay. Neurabin KO mice show a significant reduction in the level of behavioral despair in repeated FST trials at 3–5 months of age (Fig 3A), suggesting a phenotype of reduced depression-like behavior, but not at 11–13 months of age (Fig 6A). Conversely, loss of spinophilin leads to increased immobility in the first trial of FST at 3–5 months of age (Fig 3B), but a decreased level of behavioral despair after repeated FST trials at 11–13 months of age (Fig 6B). Taken together, the FST data suggest that loss of neurabin has an age-dependent protective effect against the acquisition of swimming stress-induced learned helplessness, which exists only in young adult mice and is lost with increasing age. Meanwhile, loss of spinophilin appears to only exert a protective effect against learned helplessness with increasing age. However, the apparent hyperactivity of the middle-aged spinophilin KO mice, as indicated by the OF (Fig 4B) and EZM (Fig 5B) data, is a potential confound to the findings on anxiety- and depression-related behavior. A generally higher activity level could alternatively explain the reduced FST immobility time displayed by those mice, independent of behavioral despair mechanisms. Further analysis of these aged mice will be necessary to more definitively determine whether the FST data are truly reflective of reduced depression-related behavior. Additionally, there are caveats associated with using conventional KO animals. For example, developmental abnormalities have been observed in spinophilin KO mice [26]. Striatal PP1γ expression is reduced in both spinophilin KO and neurabin KO mice [25]. These adaptive changes could contribute to behavioral alterations observed in these mice. Conditional KO mice are therefore necessary to address age- and brain region-specific functions of neurabin and spinophilin. Nevertheless, our data presented herein indicate that these two homologous proteins play important, yet distinct, roles in the regulation of anxiety- and depression-like behaviors, as well overall activity level, in an age-dependent manner. Our study therefore provides new insights into the complex neurobiology of affective disorders.
